# Dynamic BH3 profiling identifies active BH3 mimetic combinations in non-small cell lung cancer

**DOI:** 10.1038/s41419-021-04029-4

**Published:** 2021-07-27

**Authors:** Danielle S. Potter, Ruochen Du, Patrick Bhola, Raphael Bueno, Anthony Letai

**Affiliations:** 1 Dana-Farber Cancer Institute, Boston, MA 02215 USA; 2 Harvard Medical School, Boston, MA 02215 USA; 3 Brigham and Women’s Hospital, Boston, MA 02115 USA

**Keywords:** Non-small-cell lung cancer, Apoptosis, Predictive markers

## Abstract

Conventional chemotherapy is still of great utility in oncology and rationally constructing combinations with it remains a top priority. Drug-induced mitochondrial apoptotic priming, measured by dynamic BH3 profiling (DBP), has been shown in multiple cancers to identify drugs that promote apoptosis in vivo. We therefore hypothesized that we could use DBP to identify drugs that would render cancers more sensitive to conventional chemotherapy. We found that targeted agents that increased priming of non-small cell lung cancer (NSCLC) tumor cells resulted in increased sensitivity to chemotherapy in vitro. To assess whether targeted agents that increase priming might enhance the efficacy of cytotoxic agents in vivo as well, we carried out an efficacy study in a PC9 xenograft mouse model. The BH3 mimetic navitoclax, which antagonizes BCL-xL, BCL-w, and BCL-2, consistently primed NSCLC tumors in vitro and in vivo. The BH3 mimetic venetoclax, which electively antagonizes BCL-2, did not. Combining navitoclax with etoposide significantly reduced tumor burden compared to either single agent, while adding venetoclax to etoposide had no effect on tumor burden. Next, we assessed priming of primary patient NSCLC tumor cells on drugs from a clinically relevant oncology combination screen (CROCS). Results confirmed for the first time the utility of BCL-xL inhibition by navitoclax in priming primary NSCLC tumor cells and identified combinations that primed further. This is a demonstration of the principle that DBP can be used as a functional precision medicine tool to rationally construct combination drug regimens that include BH3 mimetics in solid tumors like NSCLC.

## Introduction

Lung cancer is the leading cause of cancer-related death worldwide [1]. It can be subdivided into two main histological subtypes, non-small cell lung cancer (NSCLC) and small cell lung cancer. NSCLC accounts for approximately 83% of newly diagnosed cases [2]. Since the 1970s, conventional chemotherapy has been the backbone of medical lung cancer therapy [3]. Chemotherapy regimens usually include a platinum such as cisplatin or carboplatin, combined with a taxane such as docetaxel or paclitaxel or a topoisomerase II inhibitor such as etoposide [4]. Over the past 15 years there have been advances in NSCLC therapy, particularly for patients with adenocarcinoma, with the addition of targeted agents such as gefitinib, erlotinib, and afatinib for that minority of patients with EGFR mutations or crizotinib, ceritinib, alectinib, and brigatinib for those with ALK alterations. In 2015, the FDA approved the use of PD-1 inhibitors (nivolumab and pembrolizumab) and in 2016, approved PDL-1 inhibitors (atezolizumab) in relapsed NSCLC patients [5]. In 2020, the nivolumab and ipilimumab (CTLA-4) combination was approved for first-line therapy in metastatic NSCLC [6].

Apoptosis is a form of programmed cell death [7]. The two major forms are the intrinsic and extrinsic pathway. Both ultimately result in activation of caspases resulting in proteolysis, oligonucleosomal DNA cleavage, and cell surface tagging to accelerate phagocytosis [8–10]. Intrinsic apoptosis is regulated by the BCL-2 family of proteins, which are made up of pro-apoptotic effectors (BAK, BAX, and BOK), pro-apoptotic BH3-only proteins (BIM, BID, PUMA, BAD, BMF, NOXA, and HRK), and anti-apoptotic family members (BCL-2, BCL-xL, BCL-w, MCL-1, BFL-1) [11, 12]. Commitment to death via the intrinsic pathway occurs following mitochondrial outer member permeabilization (MOMP), which causes the release of apoptotic factors such as cytochrome c from the mitochondria, which in turn form caspase-activating complexes that are required for most of the cellular phenotypes of apoptosis [13]. MOMP is a switch-like event regulated by the dynamic interactions between the pro-apoptotic and anti-apoptotic BCL-2 family members.

Mitochondrial priming, regulated by the BCL-2 family of proteins, is a measure of how close to the apoptotic threshold a cell is. A highly ‘primed’ cell will be closer to cell death and have a lower apoptotic threshold relative to a poorly primed cell which is further from cell death, with a higher apoptotic threshold. Priming can be measured with BH3 profiling, which uses BH3 peptides derived from the BH3 domain of the pro-apoptotic BH3-only family members to permeabilize mitochondria in a BAX and BAK dependent fashion. The more primed a cell is, the more sensitive its mitochondria will be to the synthetic BH3 peptide probes used in this assay [14, 15].

Perturbations can alter priming. Dynamic BH3 Profiling (DBP) is a functional tool that can measure these changes. If a drug treatment enhances mitochondrial priming then a BH3 peptide such as BIM BH3, will permeabilize the mitochondria more efficiently in drug-treated cells compared to control-treated cells [15]. Here, for the first time, we test whether targeted agents that increase the apoptotic priming of cancer cells, as measured by DBP, also enhance sensitivity of those cells to conventional chemotherapy. We propose this as a rational strategy to construct combinations of targeted therapies with conventional chemotherapy agents.

## Materials and methods

### Cell culture and drugs

A549, H522, H1975, H3255, and PC9 (also known as PC14) cells were a kind gift from Professor Caroline Dive (Cancer Research UK Manchester Institute). All NSCLC cells were cultured in RPMI media with 10% FBS, 100 U/ml penicillin and 100 µg streptomycin (Gibco) and were incubated in a humidified atmosphere at 37 °C with 5% CO2. Cell lines were authenticated using the Promega GenePrint^®^ 10 System (Promega Corporation, WI, USA; authenticated at Dana-Farber Cancer Institute (DFCI), Molecular Biology Core Facility). Fresh primary NSCLC tumor cells were cultured in HITES media (5 μg/mL insulin, 10 μg/mL transferrin, 10 nM β-estradiol, 30 nM sodium selenite and 10 nM hydrocortisone in RPMI phenol red free media) for no more than 24 h. Navitoclax, TAE684, GDC-0941 (Selleckchem), Ibrutinib, geldanamycin (ApexBio), AZD6244 (StemCell Technologies), AZD5991 (ex vivo use), A-1331852, venetoclax, S63845 (MedChemExpress), AZD5991 (in vitro use; AstraZeneca) were dissolved in DMSO (10 mmol/L; Sigma) and stored at −20 °C. For in vivo use navitoclax, S63845 and venetoclax powder was stored at −20 °C. Navitoclax was then formulated in 10% DMSO, 30% polyethylene glycol 400 (Sigma) and 60% Phosal 50 PG (American Lecithin Company) and stored at room temperature (RT) for up to 7 days. Venetoclax was formulated in 10% ethanol, 30% polyethylene glycol 400 and 60% Phosal 50 PG and stored at 4 °C for up to 5 days. S63845 was formulated in 25 mM hydrochloric acid and 20% hydroxyl-propyl-beta-cyclodextrin (Sigma) and used the same day (within 3 h).

### Drug treatment and cell viability

Cell lines were seeded at 1000 cells per well in a 384 well plate. Twenty-four hours later cells were drugged using the HP D300e digital dispenser (Hewlett-Packard). Cells were treated with chemotherapy dose–response (docetaxel 0.2–0.001 µM, etoposide 30–0.1 µM, half log fold decrease in concentration) plus the indicted concentration of targeted agent or DMSO-control for 72 h. Cell viability was measured using CellTiter Glo assay according to the manufacturer’s instructions (Promega). Luminescence relative to untreated cells (no chemotherapy) was determined, relative to each targeted agent or DMSO-control treatment for individual chemotherapy dose-response curves. We calculate etoposide or docetaxel EC_50_ plus individual targeted agent or DMSO-control (chemotherapy-only). To determine chemotherapy EC_50_, log chemotherapy drug concentration was plotted against raw luminescence, and nonlinear curve fit analysis was performed (GraphPad Prism). Statistical analysis was carried out on three independent EC_50_ readings.

Cell lines were seeded at 5000 cells per well in a 96 well plate. The next day cells were treated at indicated concentration of drug, using the HP D300e digital dispenser and 24 or 72 h later cells were trypsinized, neutralized and stained with fluorescent conjugates of Annexin-V (made according to Brumatti et al. [16]), and Hoechst in 10x Annexin-V buffer (100 mM HEPES, 40 m KCL, 1.4 M NaCl, 25 mM CaCl_2_, 7.5 mM MgCl_2_, pH 7.5). All media was collected, and cells were fixed with 4% formaldehyde and 0.5% glutaraldehyde (Electron Microscopy Sciences) made with 10x Annexin-V buffer. Cells were then analyzed by flow cytometry (BD LSRFortessa, BD). Viable cells are Annexin-V negative and Hoechst positive, and apoptotic cells % are 100 minus viable cells %. Statistical analysis was carried out on three independent apoptotic cells % readings.

### Human samples

Fresh primary NSCLC tumors obtained from tumor resections, after patients signed an informed consent approved by the Institutional Review Board (#98-063), were used for tumor dissociation to prepare a viable single-cell suspension for CROCS-HTDBP.

### Tumor dissociation

Tumors were first cut into small pieces using a scalpel and then mechanically dissociated using Miltenyi gentleMACS Dissociator with digested for 1 h at 37 °C in RPMI containing 10 mg/mL collagenase IV, 650 U/mL DNAse I and 500 U/mL hyaluronidase (Roche Diagnostics). Cells were then filtered through a 70 µm cell strainer to make a single cells solution and cell viability was assessed by trypan blue exclusion.

### DBP/high-throughput dynamic BH3 profiling (HTDBP)

Cell lines were seeded at 1000 cells per well in a 384 well plate and 24 h later treated with the indicated concentration of targeted agent using the HP D300e digital dispenser. Twenty-four hours after treatment DBP was carried out. Fresh primary NSCLC tumor cells were seeded at 5000 cells per well in collagen 1 (20 µg/cm^2^) coated 384 well plate. Two hours later primary cells were treated with CROCS using the HP D300e digital dispenser. After 24 h CROCS treatment, HTDBP was carried out. BIM BH3 peptide titration plate and DBP/HTDBP were carried out as previously described in detail by Bhola et al. [17].

### Antibodies

Nuclei were stained with Hoechst33342 (1:2000; Life Technologies) to determine a total number of cells. Cytochrome c positive cells % was measured using cytochrome c-Alexa647 antibody (#612310; 1:2000; BioLegend). A pan cytokeratin antibody (#628608; 1:1000; Biolegend) was used to identify epithelial tumor cells parent population %. BIM (C34C5) #2933S (1:1000) and Beta-Actin #4967S (1:2000) were used for Western blotting (Cell Signaling Technology).

### Imaging

All imaging was performed on the ImageXpress Micro Confocal High-Content Microscope (Molecular Devices; at the ICCB-Longwood screening facility at Harvard Medical School). A ×10 objective was used to perform all imaging.

### Image and data analysis

Image analysis was performed in MetaXpress (Molecular Devices; at the ICCB-Longwood screening facility at Harvard Medical School) using the multi-wavelength cell scoring module and the adaptive background correction module to segment cells based on an intensity above local background. This results in an approximate single-cell segmentation and the area of pan-cytokeratin or cytochrome c intensity. Cells are scored as being positive or negative based on the area. All subsequent data analysis was performed in Excel or GraphPad Prism. To determine BIM EC_50_ for DBP analysis, log BIM concentration was plotted against cytochrome c released % (100−cytochrome c positive cells %), and nonlinear curve fit analysis was performed in GraphPad Prism. Statistical analysis was carried out on three independent EC_50_ readings. CROCS treatment on primary NSCLC cells was carried out in duplicate. Delta priming % and Z-score was calculated in Excel for each treatment well in all 9 NSCLC patient samples to analyze CROCS treated HTDBP and determine hits (Z-score ≥ 3).

$$\begin{array}{ll}{Delta}\,{Priming} = {{{\% }}}\,{Positive}\,{Cytochrome}\,c\,{Cells}_{{Mean}\,{DMSO} - {control}\,{Treated}\,{Wells}}\\ \qquad\qquad\quad\quad- \,{{{\% }}}\,{Positive}\,{Cytochrome}\,c\,{Cells}_{{Drug}\,{Treated}\,{Well}}\end{array}$$

$$\begin{array}{ll}Z{ - }score = \left( {\% \,{Negative}\,{Cytochrome}\,c\,{Cells}_{{Drug}\,{Treated}\,{Well}}} \right.\\ \left. \qquad\qquad{ - \,\% \,{Negative}\,{Cytochrome}\,c\,{Cells}_{{Mean}\,{DMSO} - {control}\,{Treated}\,{Wells}}} \right)\\ \qquad\qquad \div \,{\it{{Standard}}}\,{deviation}_{{DMSO} - {control}\,{Treated}\,{Wells}}\end{array}$$

### Intracellular BH3 profiling (iBH3)

All mice were maintained within the DCFI animal facility and all experiments involving animals were conducted in accordance with the DFCI policy and animal protocol, reviewed and approved by the DFCI Institutional Animal Care and Use Committee. These mice were housed in vented caging systems in a 12 h light/12 h dark environment and maintained at uniform temperature and humidity. PC9 cell line xenografts were grown by subcutaneous injection of 5 × 10^6^ PC9 cells in 0.2 mL of 1:1 RPMI:Matrigel into the mid-dorsal flank of 8 week old female SCID-beige mice (C.B-17/IcrHsd-PrkdcscidLystbg-J; Envigo). When tumors grew to ~ 600 mm^3^ mice were randomized into 3 groups of 4 mice per group. The groups were (1) vehicle, (2) 100 mg/kg venetoclax or (3) 100 mg/kg navitoclax. Twenty-four hours after drug were administered, mice were sacrificed and tumors harvested. Tumors were dissociated as previously described. Then iBH3 was carried out as previously reported by Ryan et al. [18].

### In vivo tolerance of drug combinations

Three 8 weeks old female SCID-beige mice were housed as previously described. Mice were treated for 14 days by oral gavage with 100 mg/kg navitoclax or venetoclax, and 8 mg/kg etoposide by intraperitoneal injection on days 5, 6, and 7 then days 12, 13 and 14. For the navitoclax and S63845 tolerance study, four mice were dosed with navitoclax 100 mg/kg by oral gavage and then 8 h later, S63845 at 25 mg/kg by intravenous injection. For the duration of the dosing, mice were monitored twice daily for any changes in weight and body appearance. Monitoring was continued for a further two weeks after which the animals were sacrificed.

### In vivo efficacy of BH3 mimetic and etoposide

PC9 cell line xenografts were grown in SCID-beige mice and housed (5 mice to a cage) as previously described. Mice were monitored twice weekly for signs of tumor growth. Once a palpable tumor was detected, measurements were taken twice a week with calipers. Tumor volume was calculated using the formula 0.5 × (longest measurement) × (shortest measurement)^2^. Seven days after implantation mice bearing PC9 xenograft tumors measuring between 150 and 250 mm^3^ were randomized, using the deterministic method, into 6 groups of 7 mice, (1) vehicle only, (2) 100 mg/kg venetoclax only, (3) 100 mg/kg navitoclax only, (4) 8 mg/kg etoposide only, (5) 100 mg/kg venetoclax followed by 8 mg/kg etoposide (6) 100 mg/kg navitoclax followed by 8 mg/kg etoposide. Navitoclax and venetoclax were administered by oral gavage daily for 14 days and etoposide was administered by intraperitoneal injection on days 5, 6, and 7 then 12,13 and 14 only. Tumor measurements were continued three times a week until the tumor reached six times initial tumor volume (6xITV) after which the mouse was sacrificed.

### Statistical analysis

Statistical analysis of BIM EC_50_ Vs. Chemotherapy EC_50_ correlation was analyzed using one-tailed Spearman Rank correlation in GraphPad Prism and *p* < 0.05 was considered statistically significant. Statistical analysis of in vitro apoptotic cell % experiments was carried out using a one-way ANOVA multiple comparison, to compare the mean of each column with the mean of the DMSO-control column. This was performed in GraphPad and *p* < 0.05 was considered significant. Statistical analysis of in vitro and in vivo BIM dose response experiments was carried out using unpaired, two-tailed *t* tests to compare treated and control groups. *T*-tests were performed in Excel (Microsoft) to determine significance. *P* < 0.05 was considered statistically significant. AUC was calculated in GraphPad Prism. Statistical analysis for in vivo efficacy study tumor volume and tumor burden was carried out using a two-way ANOVA multiple comparison, comparing the means from each group in GraphPad Prism. Comparison of in vivo efficacy study survival curves used the Log-rank (Mantle–Cox) test in GraphPad Prism. *P* < 0.05 was considered statistically significant for all in vivo statistical analysis. Delta priming % correlation was analyzed using one-tailed Spearman Rank correlation in GraphPad Prism and *p* < 0.05 was considered statistically significant. Ex vivo Z-score analysis of HTDBP on CROCS treated primary NSCLC cells was used to assess a hit (≥3). Z-score was performed in Excel.

## Results

### Mitochondrial priming determines chemosensitivity in NSCLC cell lines

Targeted agents that evoke an early death signal measured by DBP have been shown to predict a cytotoxic response several days later [14, 17]. Mitochondrial priming correlates with response to chemotherapy in several disease settings [14, 19, 20]. We therefore hypothesized that targeted agents that increase mitochondrial priming, would increase the chemosensitivity of NSCLC cell lines [14, 19, 20].

**Fig. 1:**
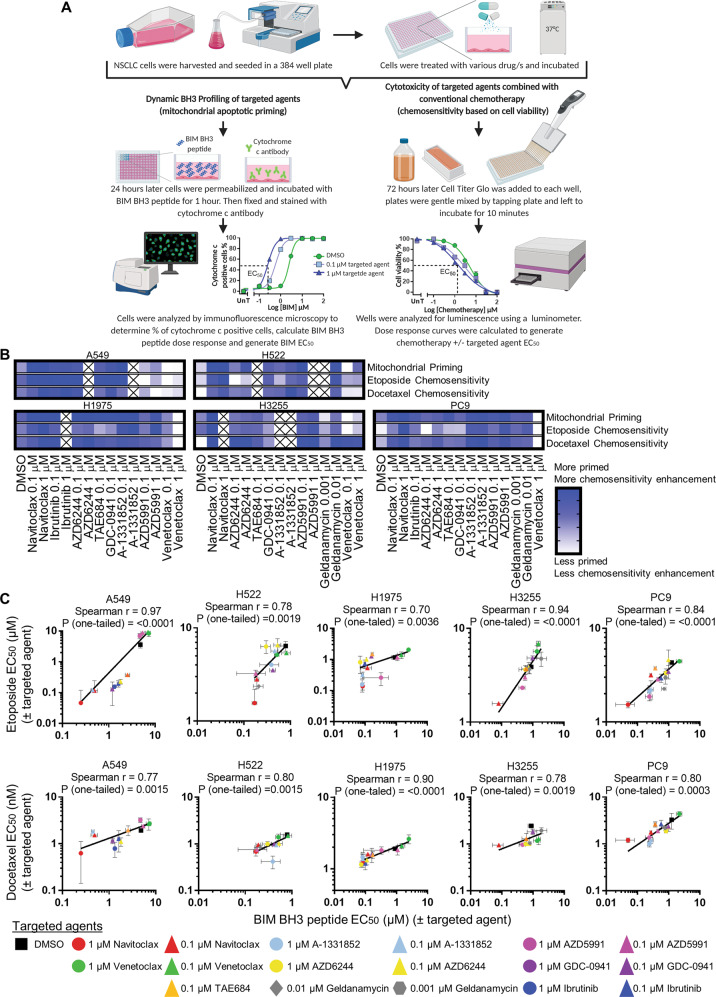
Increasing mitochondrial priming enhances NSCLC cell lines sensitivity to conventional chemotherapy. Cell lines (A549, H522, H1975, H3255 and PC9) were exposed to various targeted agents and mitochondrial priming was assessed. In parallel cells were treated to a dose response of chemotherapy ± targeted agent and chemosensitivity was assessed. **A** Schematic showing the workflow for measuring drug-induced priming using DBP and chemosensitivity using cell viability assay. Created with BioRender.com. **B** Top heat map shows normalized mitochondrial priming (BIM EC_50_) for individual targeted agents at indicated concentrations or DMSO-control. Bottom heat map shows normalized chemosensitivity to etoposide or docetaxel plus targeted agent or DMSO-control. **C** These graphs show the correlation between mitochondrial priming (BIM EC_50_) Vs. chemosensitivity (etoposide EC_50_ or docetaxel EC_50_) for each cell line for various drugs at specific concentrations or DMSO-control (either BIM, etoposide or docetaxel only). Data represents the mean BIM EC_50_, etoposide EC_50_ or docetaxel EC_50_ of three independent experiments ± standard deviation.

We treated multiple NSCLC cell lines (A549, H522, H1975, H3255, and PC9) with a panel of targeted agents and assessed whether drug-induced mitochondrial priming, measured by DBP, increases chemosensitivity (Fig. 1A). To ensure we measured mitochondrial priming (upstream of caspase activation) and not cell death, we selected concentrations of targeted agents that did not cause cell death (Fig. S1, S2). The BIM BH3 peptide promiscuously binds to all the anti-apoptotic BCL-2 family members and is a probe of overall mitochondrial priming [21, 22]. Increased priming by a drug was measured by enhanced BIM-induced MOMP, measured by release of cytochrome c from mitochondria. We incubated drug-treated cells, permeabilized with digitonin, with a range of BIM BH3 peptide concentrations for 1 h and then fixed and stained with cytochrome c antibody. Representative microscopy images for cytochrome c staining are shown in Fig. S3. We plotted the mitochondrial response to BIM BH3 to calculate the BIM EC_50_ with and without treatment with a targeted agent (Fig. 1A). We are looking at changes in mitochondrial priming and the effect on sensitivity to chemotherapy. If a targeted agent increases mitochondrial priming, then the BIM EC_50_ will be decreased compared to the DMSO-control treated wells. In Fig. 1B we show mitochondrial priming normalized to DMSO-control, following each individual drug treatment for each of the 5 NSCLC cell lines plotted in a heat map. EC_50_ data can be found in Data file S1.

In parallel we assessed the effect of the same targeted agents on the cytotoxicity of two conventional chemotherapy agents, etoposide and docetaxel (Fig. 1A). We treated each of the 5 NSCLC cell lines with a range of docetaxel or etoposide concentrations, plus the targeted agent or DMSO-control indicated in the heat map shown in Fig. 1B. We calculated the chemotherapy EC_50_ plus each of the targeted agents or DMSO-control. A reduction in docetaxel or etoposide EC_50_ plus targeted agent compared to DMSO-control (chemotherapy only) indicates an increase in chemosensitivity when combined with that targeted agent. When we compared the effect of chemosensitivity and mitochondrial priming of each targeted agent in the NSCLC cell line panel we observed a similar trend in respective heat maps (Fig. 1B). Targeted agents that increase mitochondrial priming, also increase chemosensitivity in NSCLC cell lines.

To test our directional hypothesis, whether increasing mitochondrial priming with targeted agents increased chemosensitivity in NSCLC, in Fig. 1C we tested the correlation between mitochondrial priming (BIM EC_50_, measured by DBP) and chemosensitivity (etoposide or docetaxel EC_50_, measured by cell viability) for each drug. While the magnitude of priming varied according to drug and cell line, the drug-induced change in BIM EC_50_ consistently correlated with either etoposide EC_50_ or docetaxel EC_50_ in each of the 5 NSCLC cell lines. This suggests that as mitochondrial priming is increased by targeted agents, sensitivity of NSCLC cells to chemotherapy agents is also increased.

Navitoclax (BCL-xL, BCL-2 and BCL-w antagonist) along with BCL-xL specific antagonist A-1331852, consistently primed NSCLC cells lines but venetoclax (BCL-2 antagonist) did not suggesting that it is the antagonism of BCL-xL that is priming these NSCLC tumor cells (Fig. 1). To assess if a drug that primes, such as navitoclax, combined with a chemotherapy drug such as etoposide caused apoptosis days later, we treated NSCLC cell lines with navitoclax combined with etoposide or the single agents and measure the percentage of apoptotic cells after 72 h. Navitoclax plus etoposide increased apoptosis compared to DMSO-control or either drug as a single agent **(**Fig. S4), confirming that drugs that increase priming (measured at 24-hours) combined with chemotherapy agents results in cell death days later.

To understand how navitoclax works more efficaciously with a chemotherapy agent we measured the protein levels of the pro-apoptotic protein BIM after treatment with either etoposide or docetaxel. BIM proteins levels were elevated after treatment with etoposide or docetaxel compared to DMSO-control in multiple NSCLC cell lines (Fig S5), this could explain why navitoclax plus etoposide causes more apoptosis after several days. Increased levels of BIM are buffered by anti-apoptotic BCL-2 family members. Navitoclax antagonizes the anti-apoptotic’s BCL-xL, BCL-2, and BCL-w releasing any BIM bound allowing BIM to directly activate BAK/BAX [23, 24].

### Selectivity of drug-induced mitochondrial priming is recapitulated in vivo

**Fig. 2:**
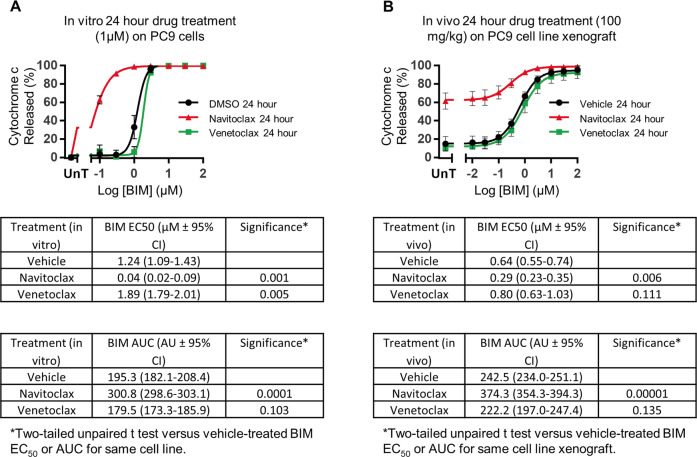
In vivo mitochondrial priming recapitulates in vitro. **A** DBP was performed on PC9 cells in vitro as described in Fig. 1**. B** Intracellular BH3 profiling was performed on PC9 tumor cells to measure in vivo mitochondrial priming after drug treatment in PC9 tumor bearing SCID-beige mice. We analyzed cells by flow cytometry for cytochrome c positive cells %. Graphs represent the means of three independent experiments ± standard deviation. Table represent the means of three independent BIM EC_50_ ± 95% confidence intervals (CI), BIM area under curve (AUC) ± 95% confidence intervals (CI) and significance is determined according to two-tailed unpaired *t* test. *P < 0.05 was considered statistically significant.

To investigate whether in vitro drug-induced mitochondrial priming response measured by DBP could be recapitulated in vivo, we generated a PC9 cell line xenograft mouse model to assess in vivo mitochondrial priming of tumors after drug treatment. The aim of this experiment was to test whether DBP accurately identified drugs that prime cancer cells in vivo. Based on the results in Fig. 1B, C for PC9 cells, we chose navitoclax as a positive and venetoclax as a negative control. Notably, Terai et al. also observed that PC9 cells are resistant to venetoclax [25]. In Fig. 2A, we show an example of the dose response curves used to generate the EC_50_ values in Fig. 1B, C. We treated PC9 cells with either 1 µM navitoclax, 1 µM venetoclax or DMSO-control for 24-h in vitro and then incubated permeabilized cells with a range of BIM concentrations to determine BIM EC_50_ and AUC (area under curve) based on cytochrome c released % (Fig. 2A). Navitoclax significantly increased mitochondrial priming, reducing the BIM EC_50_ to 0.04 µM from 1.24 µM (DMSO-control). BIM AUC increased to 300.8 AU (navitoclax) from 195.3 AU (DMSO-control). Venetoclax slightly but significantly reduced mitochondrial priming, increasing the BIM EC_50_ to 1.89 µM, compared to DMSO-control and BIM AUC was reduced to 179.5 AU.

We next tested whether targeted agents that prime in vitro will also prime tumor cells in vivo. Using the PC9 cell line xenograft model, we measured mitochondrial priming of tumor cells 24-h after mice were administered with either 100 mg/kg navitoclax, 100 mg/kg venetoclax or vehicle-control. Tumors were dissociated and the flow cytometry based BH3 profiling method was used to measure mitochondrial priming [18]. Digitonin permeabilized tumor cells were incubated with a range of BIM peptide concentrations for 1 h and then fixed and stained for cytochrome c. Cytochrome c release was plotted against BIM dose response to calculate BIM EC_50_ and AUC. Cytochrome c release observed after treatment in vivo (Fig. 2B) recapitulated response in vitro (Fig. 2A). In vivo navitoclax treatment significantly increased mitochondrial priming, reducing BIM EC_50_ to 0.29 µM compared to 0.64 µM (vehicle-control). BIM AUC increased to 374.3 AU (navitoclax) from 242.5 AU (vehicle-control). Venetoclax slightly reduced mitochondrial priming, increasing BIM EC_50_ to 0.80 µM and reducing AUC to 222.2 AU, as seen in vitro. In vivo navitoclax treatment did not just affect mitochondrial priming but also caused a significant amount of cytochrome c loss even in the absence of BIM peptide treatment (61.3%) compared to vehicle (14.7%) and venetoclax treatment (10.6%) (Fig. 2B). This suggests that our in vivo navitoclax treatment had moved beyond apoptotic priming to mitochondrial permeabilization and commitment to apoptosis [14].

### Enhancing mitochondrial priming increases chemosensitivity in vivo

We assessed the tolerability of navitoclax or venetoclax in combination with etoposide in SCID-beige mice. Mice were either treated with navitoclax and etoposide combination or venetoclax and etoposide combination daily for 14 days Fig. S6A. Both BH3 mimetics in combination with etoposide appeared to be well tolerated (Fig. S6B; Fig. S6C).

Due to the observation that drug-induced mitochondrial priming increased chemosensitivity in vitro (Fig. 1A–C), we hypothesize that drugs that increase mitochondrial priming of tumor cells would increase the efficacy of the chemotherapy when combined in vivo. PC9 xenograft mice were used to investigate the effect of the BH3 mimetics, navitoclax or venetoclax (negative control) on chemosensitivity in vivo. Once tumors reached between 150 and 250 mm^3^ mice were randomized into 6 groups; (1) vehicle-control, (2) venetoclax only, (3) navitoclax only, (4) etoposide only, (5) venetoclax + etoposide and 6) navitoclax + etoposide. Both navitoclax and venetoclax were dosed daily at 100 mg/kg for 14 days (day 1–14). Etoposide was dosed in two cycles to recapitulate the dosing NSCLC patients receive [26]. Mice were dosed with etoposide at 8 mg/kg on day 5, 6 and 7 and then again on day 12, 13 and 14.

**Fig. 3:**
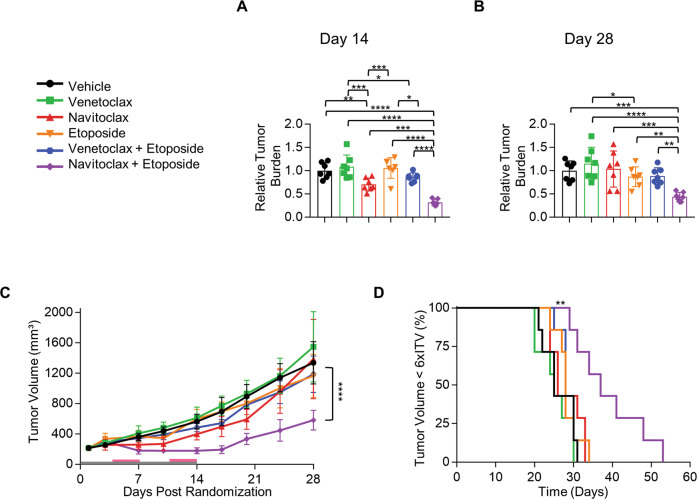
Drug-induced mitochondrial priming measured by dynamic BH3 profiling, increases chemosensitivity in vivo. Treatment groups; vehicle only (black); 100 mg/kg venetoclax only (green); 100 mg/kg navitoclax only (red); 8 mg/kg etoposide only (orange); 100 mg/kg venetoclax combined with 8 mg/kg etoposide (blue); 100 mg/kg navitoclax combined with 8 mg/kg etoposide (purple). Mice are sacrificed when tumors reached the endpoint of 6 times the initial tumor volume (6xITV). **A**, **B** Relative tumor burden calculated for each treatment group relative to the mean tumor volume of the vehicle-control group on day 14 and day 28 respectively. We calculated significance using a 2-way ANOVA multiple comparisons test, *p < 0.05, **p < 0.01, ***p < 0.001 and ****p < 0.0001. **C** Mean tumor growth over 28 days (1–14 days mice were dosed (gray bar represents continuous dosing of navitoclax, and orange bar represents etoposide dosing in two 3-day cycles), error bars represent ± standard deviation for 7 mice per treatment group. ****P < 0.0001 for navitoclax + etoposide treatment compared to all other treatment groups using a 2-way ANOVA multiple comparisons test. **D** Kaplan–Meier survival curve, mice were sacrificed at 6xITV endpoint. Navitoclax combined with etoposide treatment is statistically significant compared to every other treatment group (*p* < 0.01) based on a Log-rank (Mantle–Cox) test.

Navitoclax single agent significantly reduced tumor burden while mice were treated (day 14, vehicle-control Vs. navitoclax **p < 0.01; Fig. 3A). As soon as navitoclax was withdrawn tumors rapidly grew back comparable to vehicle-control mice (Fig. 3B). Venetoclax and etoposide as single agents or venetoclax combined with etoposide had no effect on tumor growth, tumor burden and survival, compared to vehicle-control mice (Fig. 3A–D). However, navitoclax combined with etoposide not only was efficacious while mice were treated with the combination (Fig. 3A; day 14, navitoclax + etoposide Vs. all other arms = *p* ≤ 0.001) but maintained a significant effect on tumor burden/volume compared to vehicle-control and all other arms of the study, two weeks after dosing had finished (Fig. 3B; day 28, navitoclax + etoposide Vs. all other arms = *p* ≤ 0.01; Fig. 3C; navitoclax + etoposide tumor volume Vs. all other arms = *p* ≤ 0.0001). Mouse survival was based on time taken to reach the predefined endpoint 6 times initial tumor volume (6xITV). Navitoclax combined with etoposide increased mouse survival 22 days from day 31 (vehicle-control) to day 53 (navitoclax + etoposide), which was longer compared to every other arm of the study (Fig. 3D; *p* ≤ 0.01). These data determine that navitoclax increases mitochondrial priming (Fig. 2), resulting in a decrease in tumor burden (Fig. 3A) and combining navitoclax with etoposide, results in increased chemosensitivity to etoposide (Fig. 3C). However, venetoclax does not increase mitochondrial priming and therefore has no effect on etoposide chemosensitivity (Fig. 3A–D). Thus, in vitro measurements of priming by DBP can be used to construct combination regimens that are active in vivo.

### Measuring drug-induced apoptotic priming on fresh NSCLC patient samples

**Fig. 4:**
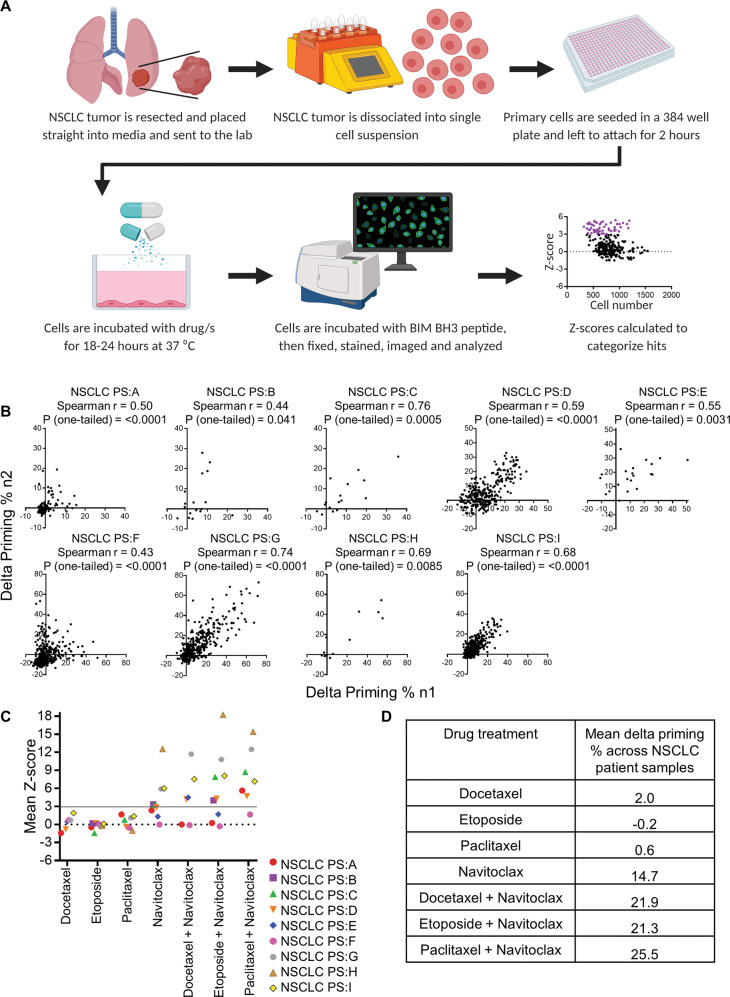
Drug-induced mitochondrial priming by navitoclax + chemotherapy compared to single agent in primary NSCLC tumor cells. Nine fresh primary NSCLC patient samples were dissociated, treated with CROCS and HTDBP carried out to identify drug/drug combinations that prime the tumor cells. **A** Schematic for CROCS-HTDBP workflow on fresh primary patient NSCLC samples. Created with BioRender.com. **B** Graph showing correlation between delta priming % replicates. **C** Graph showing mean Z-score for navitoclax and chemotherapy single agent, as well as the combination of navitoclax with chemotherapy agents. **D** Table showing mean delta priming % for navitoclax, chemotherapy single agent, and combinations in all patient samples.

Having demonstrated that enhancing priming enhanced chemosensitivity in NSCLC cell line models in vitro and in vivo, we next asked if the same principle applied to fresh human NSCLC tissue. We have previously demonstrated that drug-induced apoptotic priming in primary cancer tissues can rapidly be measured using DBP [14]. A significant advantage of DBP is that because it measures early apoptotic signaling, long term culture with a drug is not required. This means that it can be conveniently applied to patient tumor samples without requiring prolonged ex vivo culture or creation of a cell line. Primary cells can be difficult to grow long term in culture and tumor cells characteristics such as gene expression and drug sensitivity change as cells are grown on plastic [27] or passaged in vivo [17, 28]. HTDBP was developed to investigate many drug conditions simultaneously, to assess drug/s that prime the tumor cell and therefore are likely efficacious therapy for patients [17]. This approach on NSCLC patient samples is shown in Fig. 4A. While HTDBP has the capacity to test thousands of drugs simultaneously, given limiting patient tissue in this context, we focused on drugs (and their combinations) clinically relevant to NSCLC (Table S1). This clinically relevant oncology combination screen (CROCS) includes navitoclax, AZD-5991 (MCL-1 antagonist), and other conventional and targeted agents. A BCL-xL antagonist was not included in this drug list because to date one has never made it into clinical trials. We wanted to focus on clinically relevant drugs with the rational that drug may take less time to be combined in the clinic. Unfortunately, because of the different solvent requirements of cisplatin and carboplatin, they were incompatible with this approach as drugs must be in DMSO.

To first treat NSCLC primary tumor cells with CROCS and then carry out HTDBP to identify hits, we must determine the optimum BIM concentration to use in HTDBP assay. This is the point where MOMP is about to occur in untreated cells (EC_10_), so that the effects of drug induction, of additional apoptotic signaling can be more sensitively captured. We received resected, chemotherapy naïve, NSCLC patient tumors and dissociated to produce a single cell suspension. NSCLC primary tumor cells were seeded and the next day we carried out a BIM titration on untreated NSCLC primary tumor cells to calculate BIM EC_10_ (Fig. S7), then HTDBP on CROCS-treated NSCLC primary tumor cells. NSCLC patient disease subtype, tumor size received, and cellularity are shown in Table S2. For the CROCS each drug/s (single agent and combinations) is placed in duplicate. To test repeatability, we tested the correlation between experimental duplicates (delta priming % n1 Vs. delta priming % n2). Delta priming % equals the mean cytochrome c positive cells % for DMSO-control wells minus the cytochrome c positive cells % for drug-treated well [14, 17]. All 9 NSCLC patient samples showed significant correlation between delta priming % replicates (Fig. 4B), even when the number of drugs used was low (Table S3).

### Navitoclax enhances apoptotic priming in combination with conventional chemotherapy in primary NSCLC tumors

Above we showed that navitoclax enhanced apoptotic priming in NSCLC cell lines, increasing killing by conventional chemotherapy agents. Therefore, we next asked whether adding navitoclax to conventional agents increased apoptotic priming in primary NSCLC samples. As part of CROCS-HTDBP, we included treatment of cells with navitoclax alone, chemotherapy alone (etoposide, paclitaxel or docetaxel) or navitoclax in combination with chemotherapy agent. A Z-score ≥ 3 is a positive hit in our screen as it means the BIM-induced cytochrome c released % is ≥ 3 standard deviations from the mean DMSO-control wells. Mean Z-score for chemotherapy single agent, navitoclax single agent and navitoclax plus chemotherapy combination is plotted for 9 NSCLC patient samples (Fig. 4C). Note that none of the conventional agents’ docetaxel, etoposide or paclitaxel, used singly, induce priming ≥ a Z-score of 3. Single agent navitoclax primes most samples with a Z-score ≥ 3 (6/9; Fig. 4C). Mitochondrial priming is further enhanced when navitoclax is combined with a chemotherapy agent (Fig. 4C, D). These results suggest that HTDBP can generally identify combinations that can enhance apoptotic signaling in NSCLC. More specifically, these results suggest that navitoclax or other BCL-XL antagonists might have broad activity in NSCLC in combination with agents already in use.

### BH3 mimetics dominate effective drug combinations in primary NSCLC tumors

**Fig. 5:**
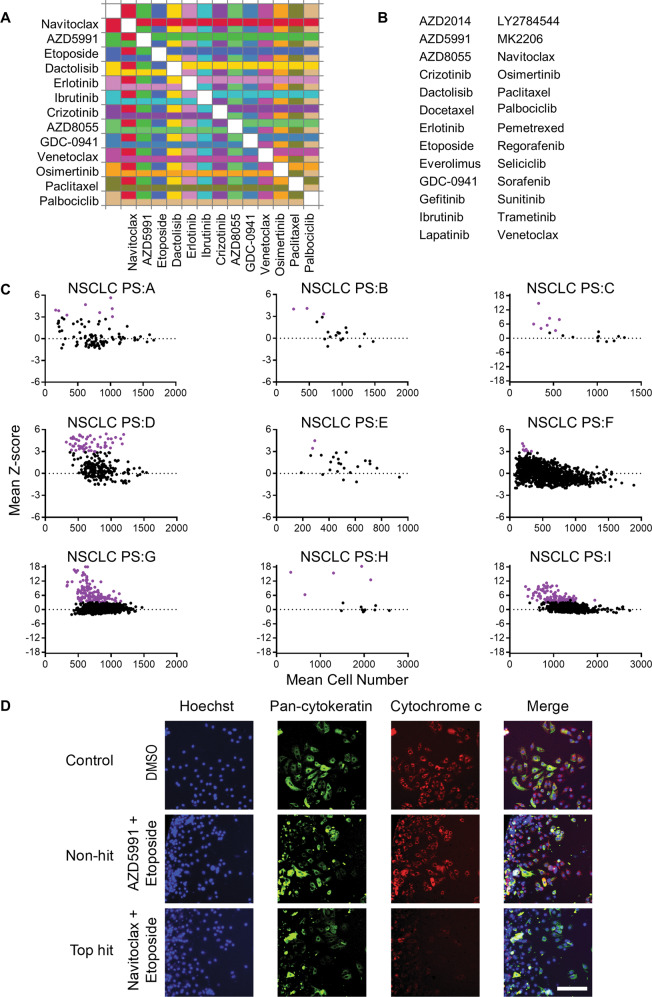
CROCS on fresh primary NSCLC tumor cells using HTDBP to identify hits. **A** An example of the layout used for CROCS treatment with 13 drugs. Each drug is dispensed horizontally, and each drug is dispensed vertically using the HP D300e digital drug dispenser. **B** Alphabetical list of all the possible drugs used in CROCS. **C** Graphs show mean Z-score Vs. mean cell number for each of the 9 primary patient NSCLC samples. Each dot represents a different drug treatment (single agent or drug-drug combination). Each drug treatment is carried out in duplicate. A purple dot represents a hit with a Z-score ≥ 3. Black dots are non-hits below the hit threshold (<3). **D** Representative immunofluorescence microscopy images from patient sample PS:H. Images taken at 10-fold magnification. Scale bar is 100 μm. Hoechst 33342 used to stain DNA (blue) and therefore identify the number of cells present in each well. Pan-cytokeratin antibody used to identify epithelial/tumor cells (parent population). From the parent population the cytochrome c positive cells % was determined using cytochrome c-647 antibody (red). DMSO treatment is a negative control for cytochrome c loss. Non-hit is a drug treatment that didn’t score a Z-score ≥ 3. Hit is the drug treatment that scored Z-score ≥ 3. The top hit is the highest Z-score for that primary NSCLC sample.

Combinations can improve response rates even without synergy or additive effect, as multiple drugs reduce the chances of a tumor being resistant to all in the combination [29]. We therefore wanted to use HTDBP to identify additional drug combinations that enhance apoptotic signaling in NSCLC primary tumors. An example of the CROCS layout is shown in Fig. 5A and total possible drug list in Fig. 5B with drug targets found in Table S1. The NSCLC tumor cell count determined the number of drugs used in CROCS (Table S3). Primary NSCLC tumor cells were treated with CROCS and HTDBP was carried out ~20 h later to identify drug/s that prime primary NSCLC tumors. For each NSCLC patient sample the drug treatments mean Z-score was plotted vs. mean cell number. Hits are shown in purple (Z-score ≥ 3; Fig. 5C).

Top common hits from all NSCLC patient samples are shown in Table 1. All the hits for each patient sample can be found in Data file S2. Top two hits were either navitoclax or AZD5991 in combination with paclitaxel. Another of the top hits is navitoclax in combination with AZD5991. This combination blocks the anti-apoptotic’s, BCL-2, BCL-xL and BCL-w in combination with MCL-1. We did a tolerance study in mice, of navitoclax and S63845 (MCL-1 antagonist) at doses well-tolerated as single agents (Fig. S8). Unfortunately, all the mice in the navitoclax and S63845 combination arm died within 4 h of treatment.

**Table 1:** NSCLC patient samples top common drug hits.

Ranked	Drug/s top hit	# patient samples with hit	# patient samples with drug/s included in CROCS	% of patients with hit
1	AZD5991/Paclitaxel	7	7	100
2	Navitoclax/Paclitaxel	6	7	85.7
3	AZD5991/AZD8055	4	5	80
4	AZD5991/Crizotinib	4	5	80
5	Navitoclax/AZD5991	7	9	77.8
6	Navitoclax/Venetoclax	3	4	75
7	AZD5991/Gefitinib	3	4	75
8	AZD5991/Trametinib	3	4	75
9	Navitoclax/Lapatinib	3	4	75
10	Navitoclax/Trametinib	3	4	75
11	Etoposide/Navitoclax	6	9	66.7
12	Navitoclax	6	9	66.7
13	Docetaxel/Navitoclax	4	6	66.7
14	Navitoclax/Crizotinib	3	5	60
15	Navitoclax/MK-2206	3	5	60
16	Navitoclax/Sorafenib	3	5	60
17	AZD5991/Ibrutinib	3	5	60
18	Navitoclax/LY2784544	3	5	60
19	AZD5991/LY2784544	3	5	60
20	AZD5991/MK-2206	3	5	60
21	Navitoclax/Ibrutinib	3	5	60
22	Navitoclax/Osimertinib	4	8	50
23	AZD5991/Docetaxel	3	6	50
24	Navitoclax/GDC-0941	3	7	42.9
25	AZD5991/GDC-0941	3	7	42.9

All the top scoring drug treatments (>40% of patient samples had the hit), contained either navitoclax and/or AZD5991, highlighting the utility of BH3 mimetics in priming primary NSCLC tumors in conjunction with clinically relevant agents. Oncopanel testing was carried out on 7 of the 9 patient samples. With the exception of patient sample E and I which had KRAS mutations, none bear mutations of known clinical significance (Data file S3) [30]. Representative images for a top hit and a non-hit in primary NSCLC tumor cells are shown in Fig. 5D, S9. Clinical information on what therapy each patient received after surgery and their overall response is very limited, so we are unable to draw any conclusions based on comparing clinical data with our findings (Table S4).

## Discussion

**Fig. 6:**
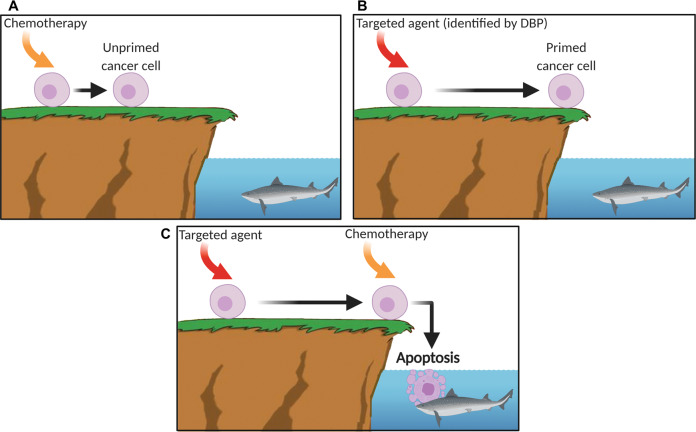
Drug-induced priming increases chemosensitivity resulting in apoptosis. Mitochondrial outer membrane permeabilization (MOMP) is a switch-like event regulated by the BCL-2 family of proteins. One can imagine MOMP like a cliff and proximity to the cliffs edge has physiological consequences such as commitment to cell death following a toxic perturbation. We consider cells that are relatively close to the cliff’s edge to be relatively “primed” for apoptosis, whereas those relatively far from the cliff’s edge to be relatively “unprimed” In comparison. **A** When an unprimed cancer cell receives a proapoptotic signal from a chemotherapy agent, it may move a small bit closer to the edge but is not compelled to commit to apoptosis. **B** A targeted agent, identified by DBP, that primes the cancer cell, pushes it closer to the cliffs edge and therefore lowering the apoptotic threshold. **C** Drug-induced priming lowers the apoptotic threshold, so the cell is more sensitive to the chemotherapy agent resulting in apoptosis. The key will be to identify the right targeted agent that primes the tumor cell for that patient and combine it with chemotherapy to increase chemosensitivity. Created with BioRender.com.

Despite appropriate enthusiasm for targeted therapies, conventional chemotherapy continues to be an important tool in oncology. Millions of cancer patients have been cured with conventional chemotherapy. Conventional chemotherapy even today continues to be the foundation of nearly all curative regimens. In this paper we outlined a generally applicable way of making cancer cells more sensitive to conventional chemotherapy. We propose that such functional methods could be systematically applied for the identification of combination regimes with conventional chemotherapy, perhaps even in a personalized way (Fig. 6).

There have been therapeutic advances over the last 15 years in NSCLC with addition of targeted agents and immunotherapies to current treatment options. Patients with wild-type ALK or EGFR along with no PDL-1 expression have a more limited therapeutic portfolio and usually get standard of care chemotherapy regimens. Identifying the most efficacious drug/s for individual patients is ideal but not always possible if the patient’s tumor does not bear an actionable predictive biomarker. We have shown that DBP can be used as a functional biomarker to identify targeted agents that enhance mitochondrial priming of cancer cells even without a genetically driven target. Targeted agents that prime could then be combined with standard of care chemotherapy regimens to increase chemosensitivity. DBP could be a valuable tool to overcome imperfect knowledge of what patient should receive what therapy.

It is often asked how we can construct active combinations that include BH3 mimetics in solid tumors. Our results suggest both BCL-xL and MCL-1 antagonism in combination with standard of care chemotherapy regimens should be further investigated in NSCLC. Note that platelet sparing BCL-xL antagonism strategies may become available in the clinic [31]. The choice between BCL-xL and MCL-1 antagonism might best be made in a personalized way, guided by a functional predictive biomarker like DBP. Indeed, since navitoclax combined with AZD5991 was a top hit, it may be that a choice between the two is not ultimately necessary if tolerable clinical combinations of the two can be identified. Experience with BH3 mimetic antagonism of anti-apoptotic proteins is greatest in chronic lymphocytic leukemia and acute myelogenous leukemia. A lesson learned in both those diseases is that the right BH3 mimetic, perhaps chosen via BH3 profiling, can make the drugs that already work in a specific context, work better. Our results here suggest that that lesson is applicable to solid tumors as well. They furthermore indicate that in NSCLC, the right BH3 mimetic is one that antagonizes BCL-xL and/or MCL-1 rather than BCL-2.

## Supplementary information

Figure S1: Targeted agent dose response curves in NSCLC cell lines.

Figure S2: Cell death does not occur at drug concentrations used to measure mitochondrial priming in vitro.

Figure S3: Representative dynamic BH3 profiling microscopy images of NSCLC cell lines.

Figure S4: Navitoclax plus etoposide increases apoptosis compared to either drug as a single agent in NSCLC.

Figure S5: BIM protein levels after treatment with etoposide or docetaxel in NSCLC cell lines.

Figure S6: BH3 mimetics combined with etoposide are well tolerated in vivo.

Figure S7: BIM dose response on fresh primary NSCLC tumor cells to calculate BIM EC10.

Figure S8: Navitoclax or S63845 single agent are well tolerated in vivo but the navitoclax and S63845 combination is not.

Figure S9: Representative CROCS-HTDBP microscopy images of primary NSCLC tumor cells.

Supplementary Figure legends

Table S1: CROCS drug list, mechanism of action and clinical status

Table S2: NSCLC patient sample disease subtype, tumor weight and tumor cellularity

Table S3: NSCLC patient sample characterization

Table S4: NSCLC patient samples clinical data

Data file S1: EC50 data for Figure 1

Data file S2: All NSCLC patients sample hits

Data file S3: Oncopanel results

## Data Availability

All data needed to evaluate the conclusions in the paper are present in the paper or the Supplementary Materials. In vitro use of AZD5991 was used under an MTA with Astra-Zeneca.
