# Supplementary figures and images for: Dynamic BH3 profiling identifies active BH3 mimetic combinations in non-small cell lung cancer
Source: Cell Death Dis. 2021 Jul 27;12(8):741. doi: 10.1038/s41419-021-04029-4 (PMC8316436; doi:10.1038/s41419-021-04029-4)

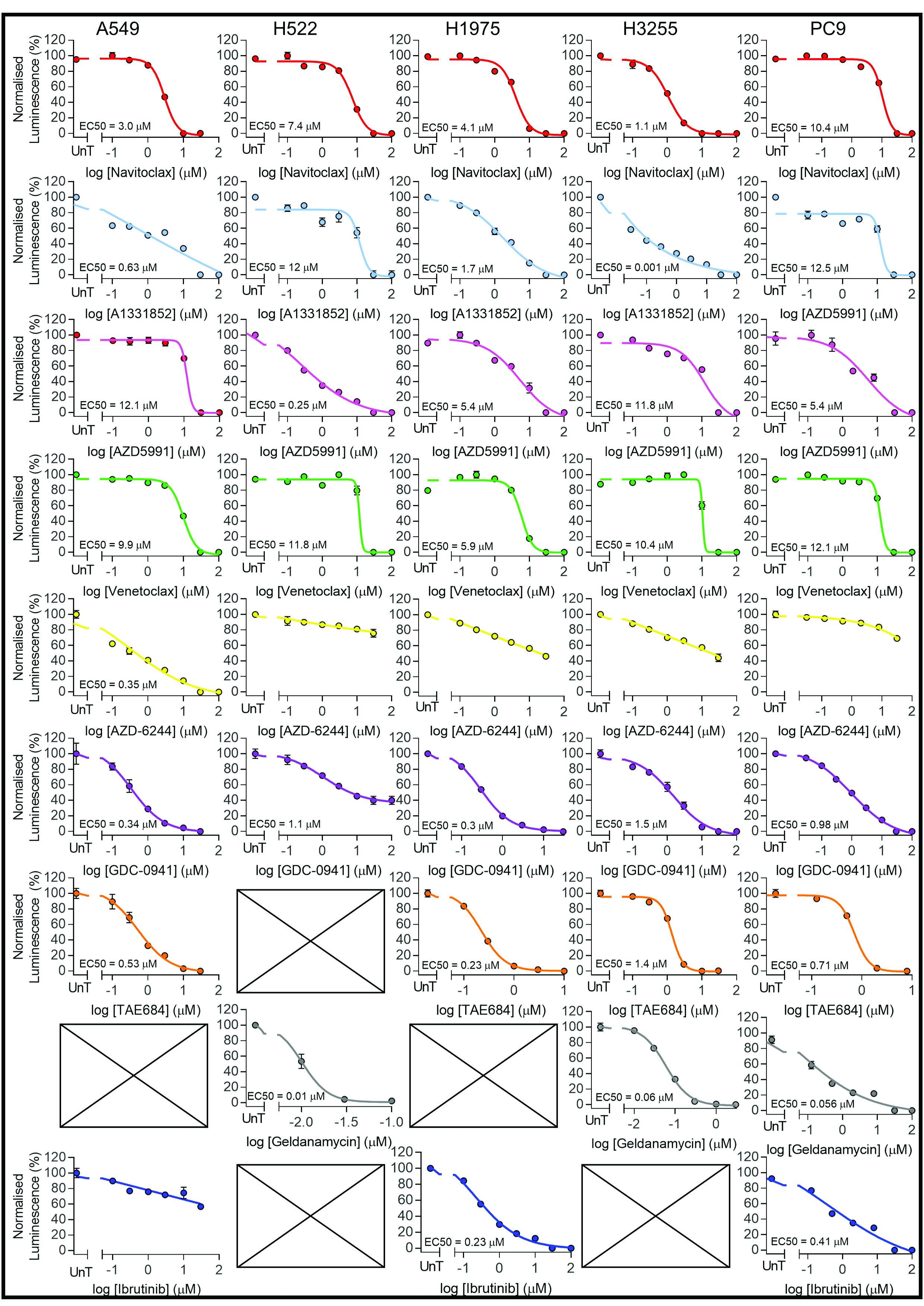

Supplement: Supplementary file 1 — Figure S1: Targeted agent dose response curves in NSCLC cell lines. [file 41419_2021_4029_MOESM1_ESM.tif]

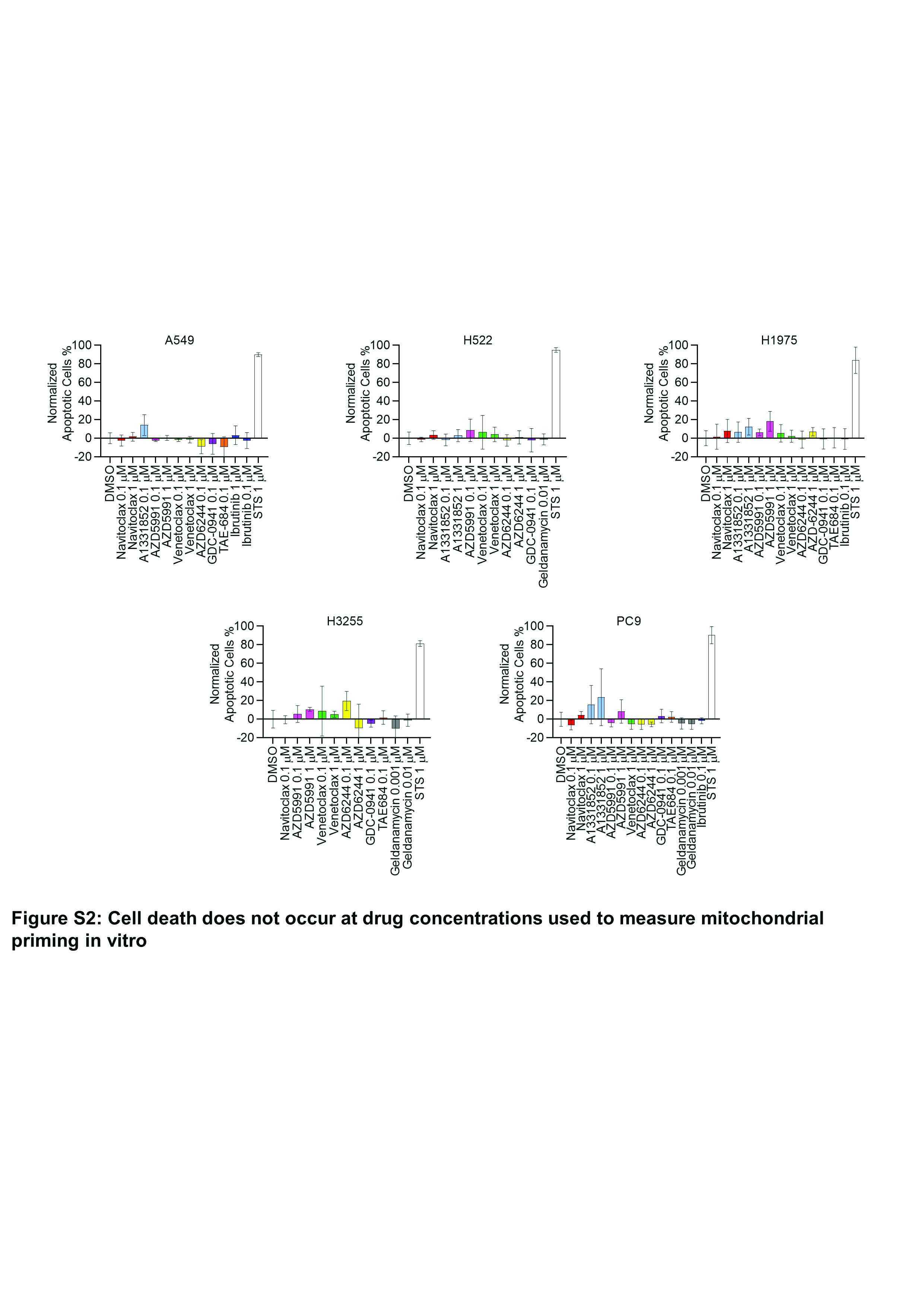

Supplement: Supplementary file 2 — Figure S2: Cell death does not occur at drug concentrations used to measure mitochondrial priming in vitro. [file 41419_2021_4029_MOESM2_ESM.tif]

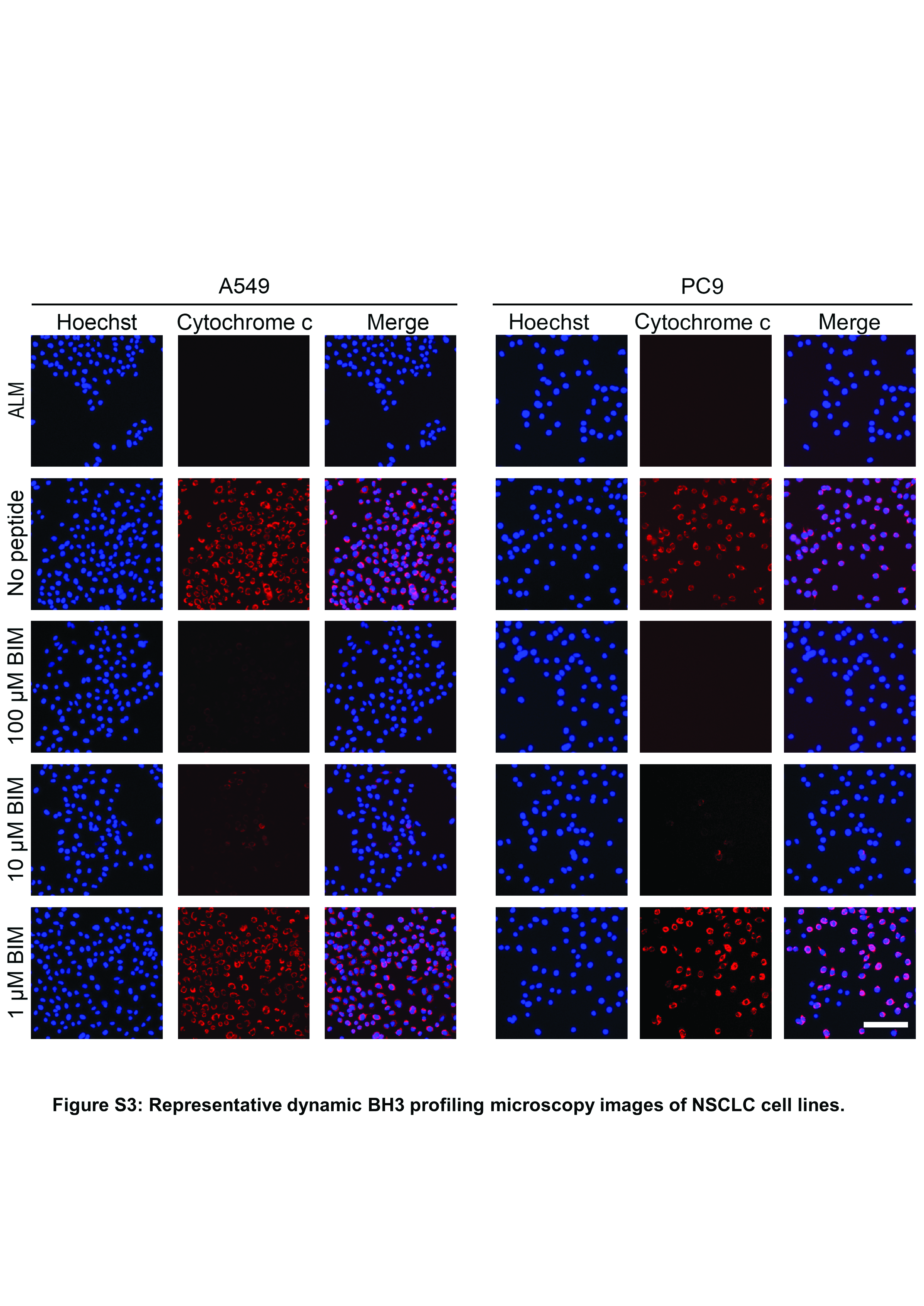

Supplement: Supplementary file 3 — Figure S3: Representative dynamic BH3 profiling microscopy images of NSCLC cell lines. [file 41419_2021_4029_MOESM3_ESM.tif]

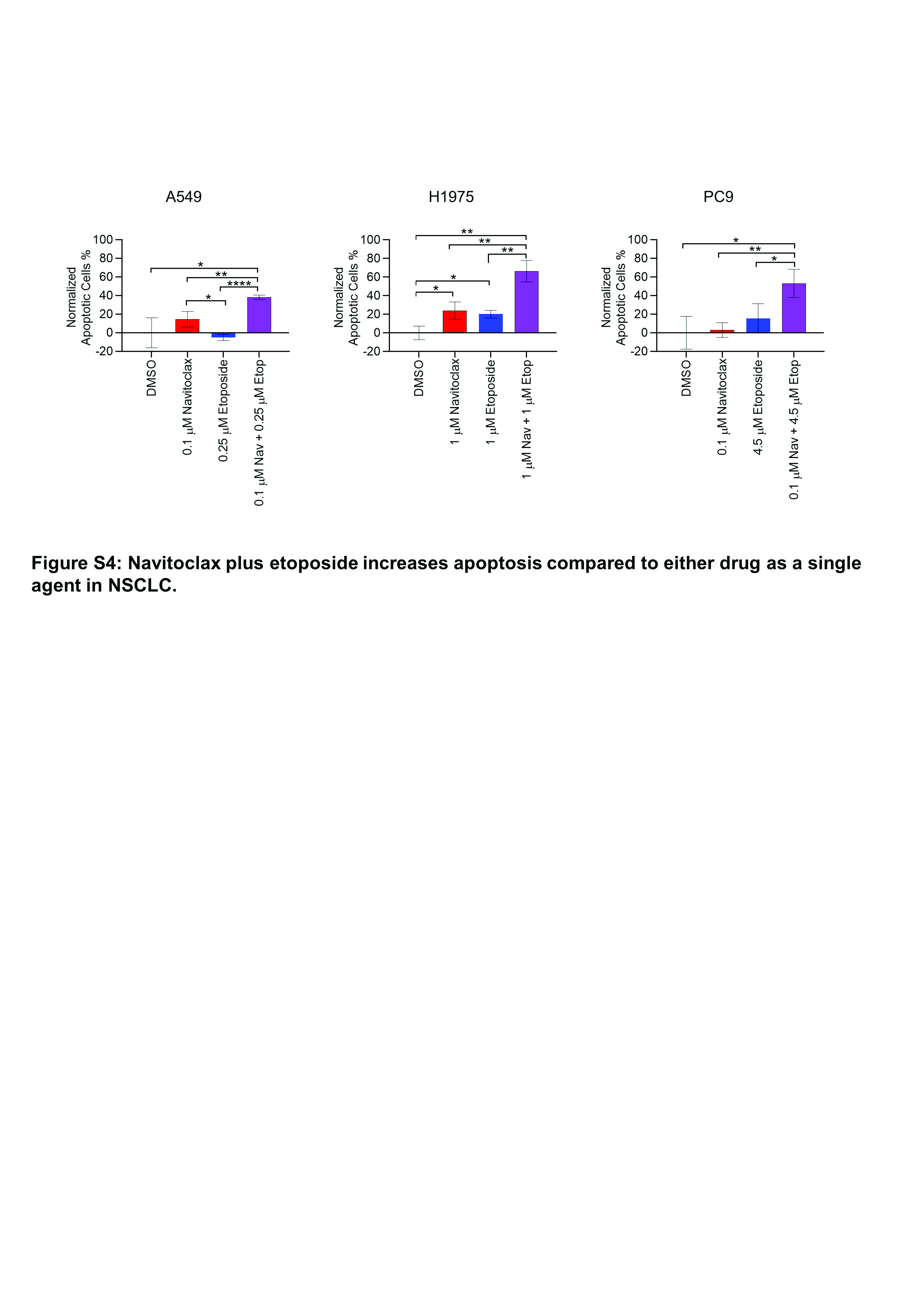

Supplement: Supplementary file 4 — Figure S4: Navitoclax plus etoposide increases apoptosis compared to either drug as a single agent in NSCLC. [file 41419_2021_4029_MOESM4_ESM.tif]

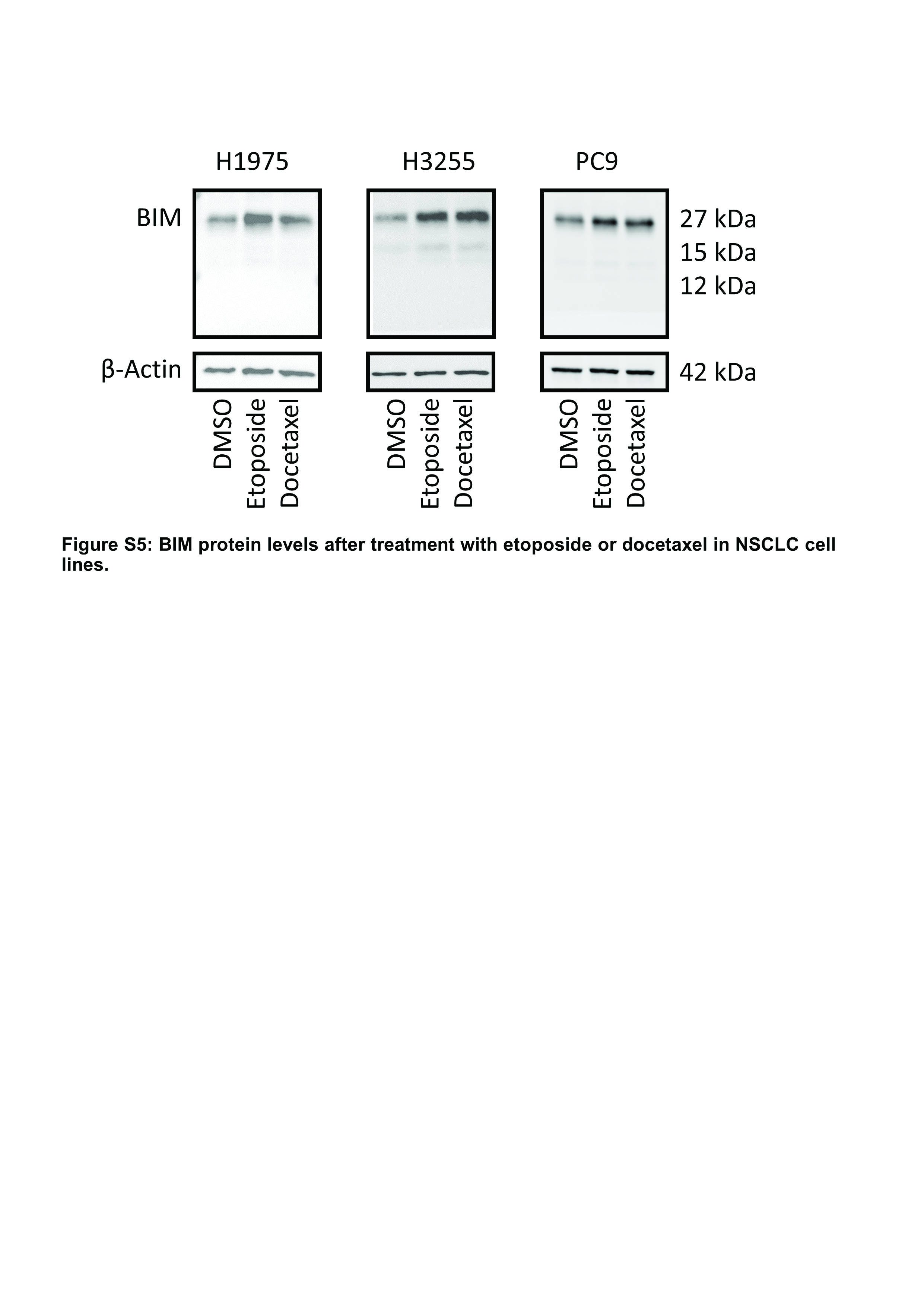

Supplement: Supplementary file 5 — Figure S5: BIM protein levels after treatment with etoposide or docetaxel in NSCLC cell lines. [file 41419_2021_4029_MOESM5_ESM.tif]

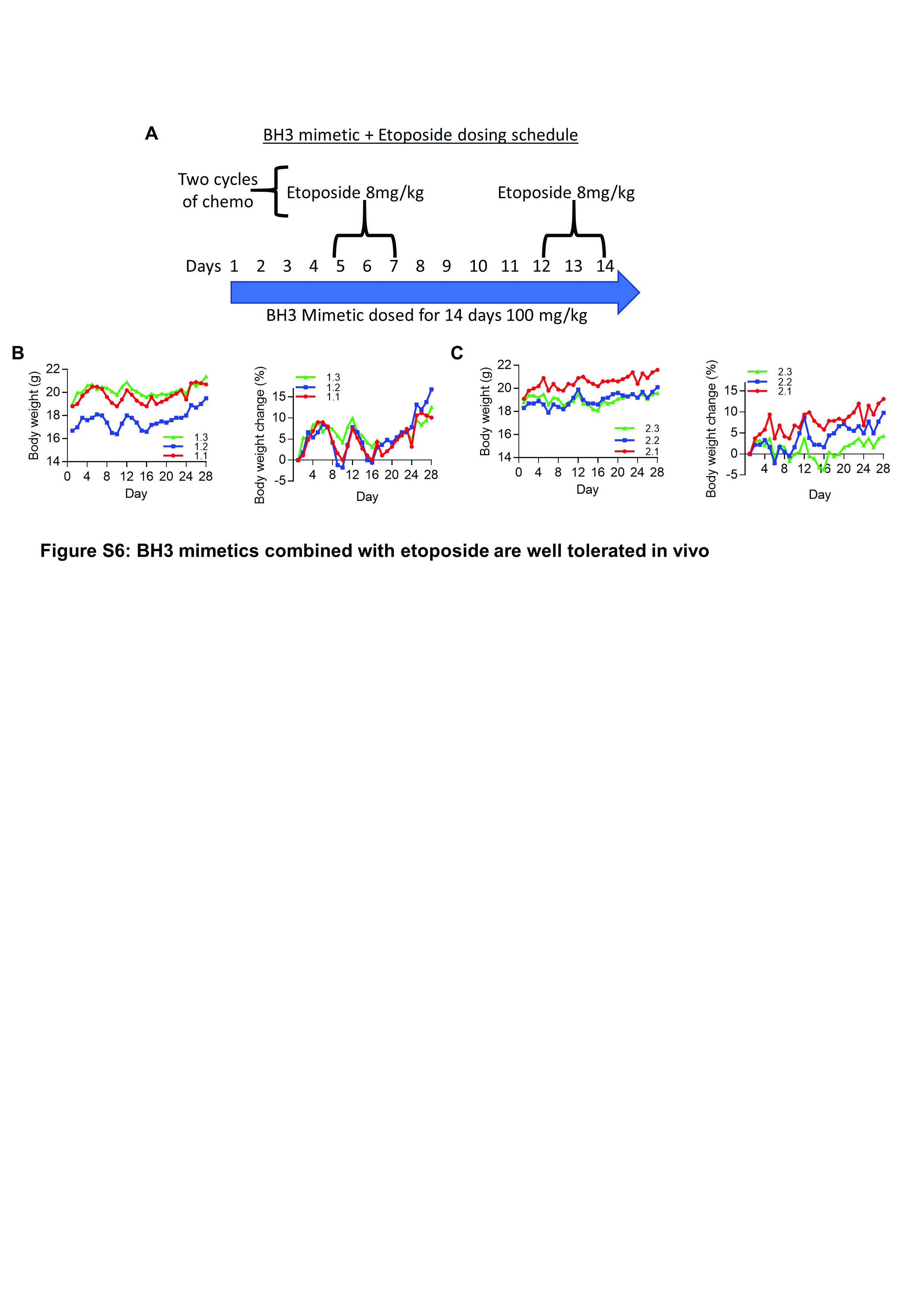

Supplement: Supplementary file 6 — Figure S6: BH3 mimetics combined with etoposide are well tolerated in vivo. [file 41419_2021_4029_MOESM6_ESM.tif]

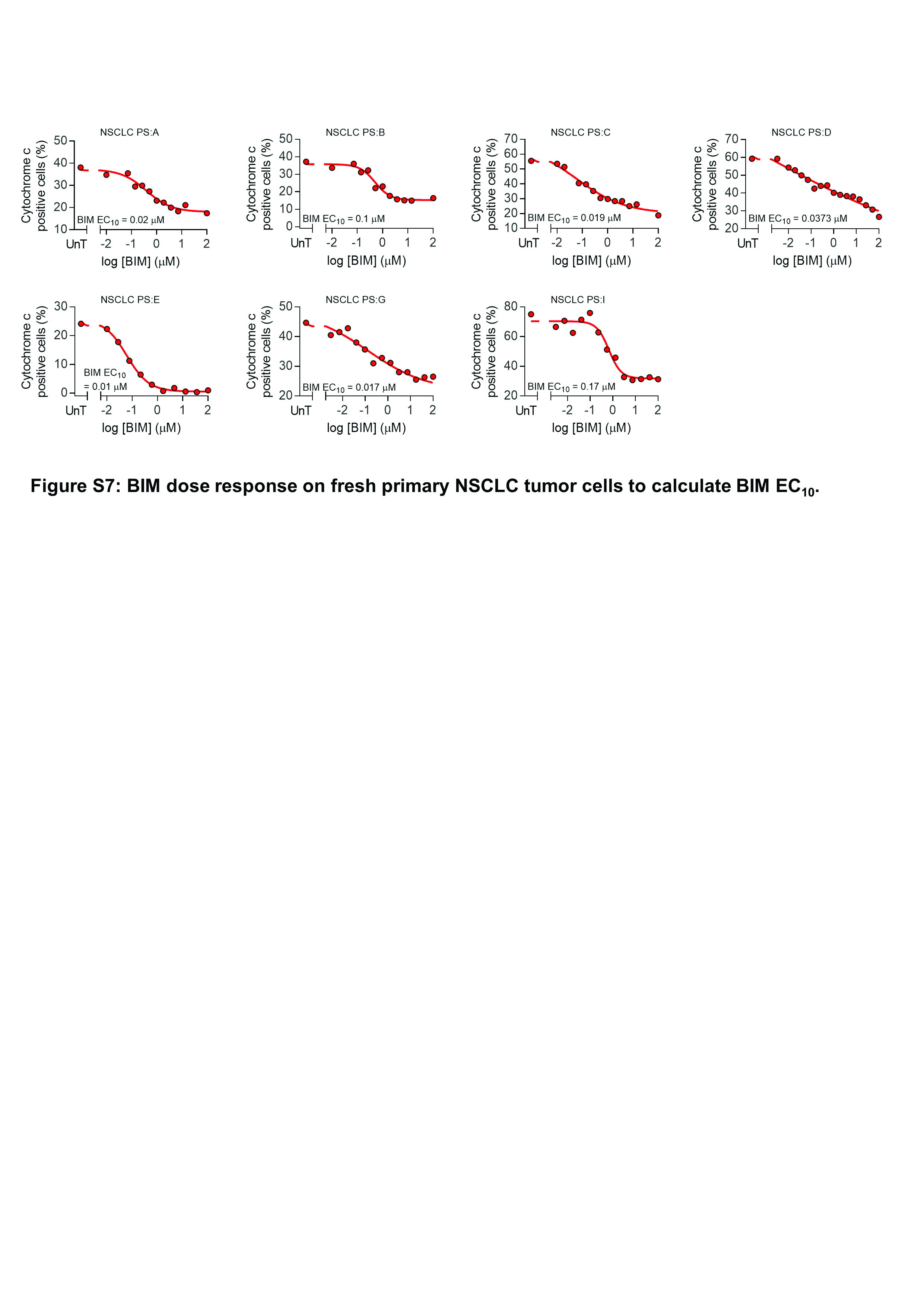

Supplement: Supplementary file 7 — Figure S7: BIM dose response on fresh primary NSCLC tumor cells to calculate BIM EC10. [file 41419_2021_4029_MOESM7_ESM.tif]

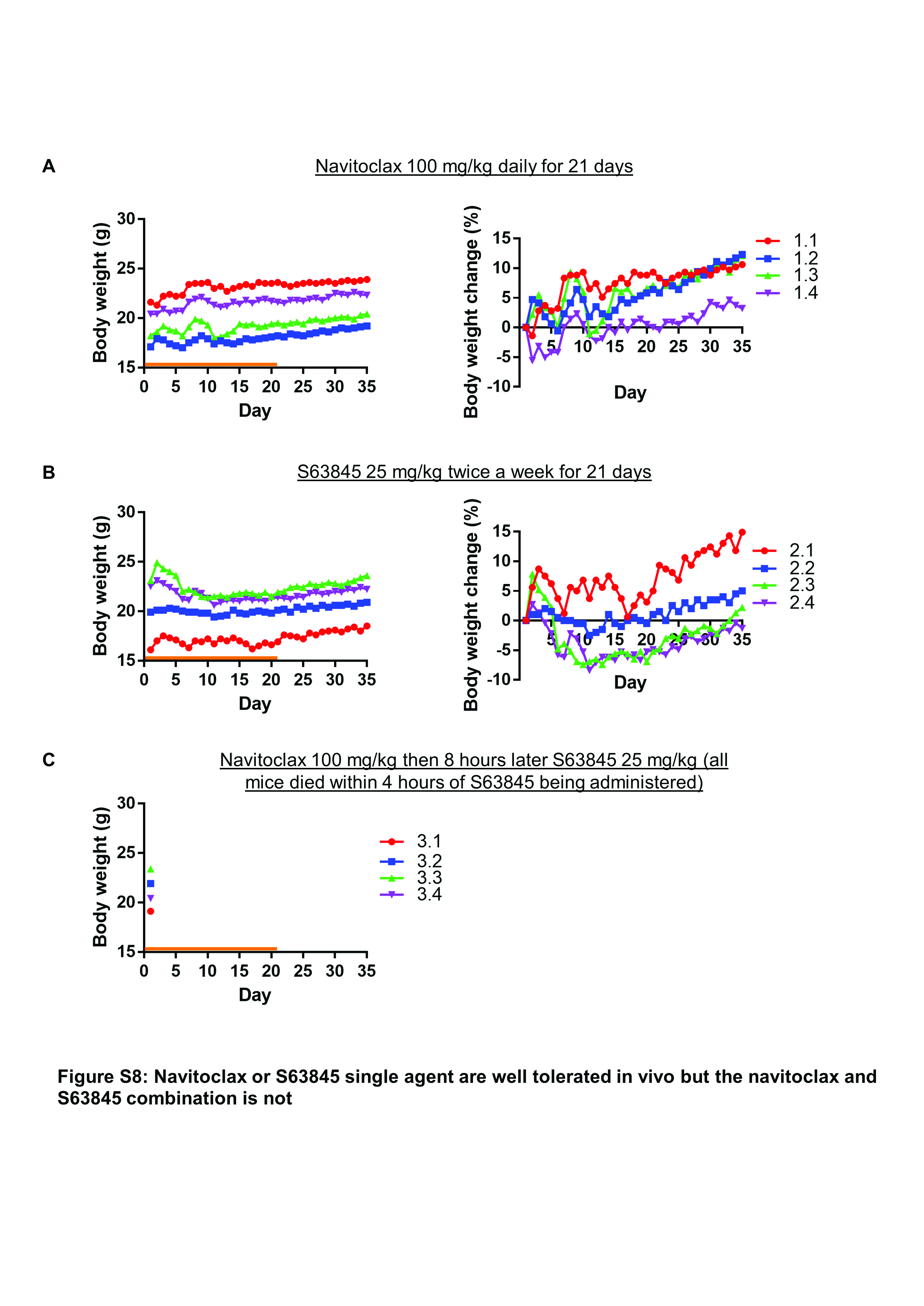

Supplement: Supplementary file 8 — Figure S8: Navitoclax or S63845 single agent are well tolerated in vivo but the navitoclax and S63845 combination is not. [file 41419_2021_4029_MOESM8_ESM.tif]

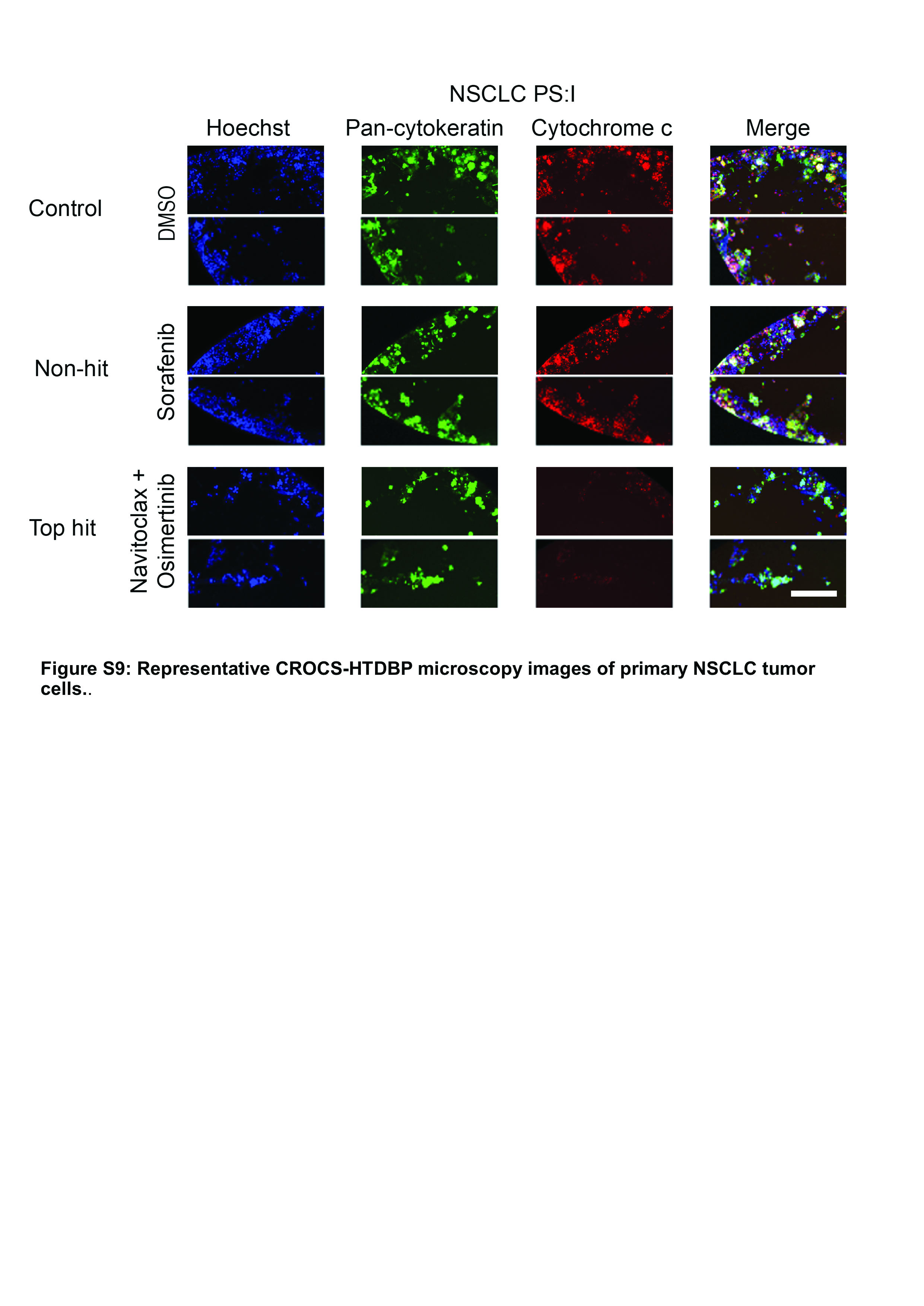

Supplement: Supplementary file 9 — Figure S9: Representative CROCS-HTDBP microscopy images of primary NSCLC tumor cells. [file 41419_2021_4029_MOESM9_ESM.tif]
