# Supplementary material for: Dynamic BH3 profiling identifies active BH3 mimetic combinations in non-small cell lung cancer
Source: Cell Death Dis. 2021 Jul 27;12(8):741. doi: 10.1038/s41419-021-04029-4 (PMC8316436; doi:10.1038/s41419-021-04029-4)
Supplement: Supplementary file 10 — Supplementary Figure legends [file 41419_2021_4029_MOESM10_ESM.docx]

**Supplementary Figure legends**

**Figure S1: Targeted agent dose response curves in NSCLC cell lines**. NSCLC cell lines (H522, H3255, PC9, A547 and H1975) were exposed with the indicated concentration of drug for 72 hours. Cell viability was assessed using Cell Titer Glo. Graph represents three independent experiments ± standard deviation.

**Figure S2: Cell death does not occur at drug concentrations used to measure mitochondrial priming in vitro.** Cells were exposed to the indicated concentration of targeted agent for 24 hours and Annexin V positive cells % were measured to determine apoptotic cells %. Bar graph showing apoptotic cells % for each treatment. 1 µM staurosporine (STS) is used as a positive control. None of the drug treatments (excluding the positive control) were statistically different to DMSO-control wells based on a one-way ANOVA multiple comparison test. Treatments were normalized to mean of DMSO-control wells. Graph represents three independent experiments ± standard deviation.

**Figure S3: Representative dynamic BH3 profiling microscopy images of NSCLC cell lines.** A549 and PC9 NSCLC cell lines were treated as previously described in **Fig. 1**. Immunofluorescence microscopy images of A549 and PC9 cell lines were taken at 10-fold magnification. Hoechst 33342 was used to stain DNA (blue) and therefore identify the number of cells present in each well (parent population). From the parent population the cytochrome c positive cell % was determined using cytochrome c-647 antibody (red). Alamethicin (ALM) was used as a positive control and no peptide was used as a negative control for cytochrome c loss. BIM peptide concentration used at indicated concentration on the left. Scale bar is 100 um.

**Figure S4: Navitoclax plus etoposide increases apoptosis compared to either drug as a single agent in NSCLC.** Cells were exposed to the indicated concentration of navitoclax, etoposide or the combination for 72 hours and Annexin V positive cells % were measured by flow cytometry to determine apoptotic cells %. Apoptotic cells were normalized to DMSO-control wells. Bar graph showing apoptotic cells % for each treatment. *P < .05, **P < .01, and ***P < .001 according to two-tailed unpaired t test. Graph represents the mean of three independent experiments ± standard deviation.

**Figure S5: BIM protein levels after treatment with etoposide or docetaxel in NSCLC cell lines.** H1975, H3255 and PC9 cells were treated with EC_25_ of etoposide (H1975 0.2 µM; H3255 1.4 µM; PC9 1.8 µM) or docetaxel (H1975 1 nM; H3255 1.3 nM; PC9 2.6 nM) for 24 hours and then cells were lysed for western blotting the pro-apoptotic BIM protein. This Figures is representative of n=3.

**Figure S6: BH3 mimetics combined with etoposide are well tolerated in vivo**. **(A)** Schematic dosing schedule for navitoclax or venetoclax in combination with etoposide over 14 days of dosing. **(B & C)** A tolerance study was carried out on the host mouse, SCID-beige. Treatment was with either navitoclax (**B**; 3 mice) or venetoclax (**C**; 3 mice) daily for 14 days and etoposide on days 5, 6 and 7, then days 12, 13, and 14. Body weight/change (based on day 0 body weight) was monitored for 28 days.

**Figure S7: BIM dose response on fresh primary NSCLC tumor cells to calculate BIM EC_10_.** A BIM dose response on untreated NSCLC primary tumor cells was carried to calculate the optimum BIM concentration where MOMP is about to occur (BIM EC_10_). The calculated BIM EC_10_ is then used in HTDBP.

**Figure S8: Navitoclax or S63845 single agent are well tolerated in vivo but the navitoclax and S63845 combination is not.** A tolerance study was carried out on the host mouse, SCID-beige and mouse weight and weight change was measured daily for 35 days (21 days of dosing and 14 days recovery period after dosing). Treatment was with either navitoclax (A; 4 mice), S63845 (B; 4 mice) or navitoclax + S63845 combination (C) for 21 days. Navitoclax was dosed at 100 mg/kg by oral gavage daily for 21 days. S63845 was dosed at 25 mg/k by intravenous injection, twice a week (Tuesday and Friday) for 21 days. The combination was scheduled to be dosed at the same concentration as the single agent arms with navitoclax dosed first and S63845 dosed 8 hours later. Unfortunately, all mice in the navitoclax and S63845 combination arm died withing 4 hours of the second drug S63845 being administered. Mice died of serve toxic events.

**Figure S9: Representative CROCS-HTDBP microscopy images of primary NSCLC tumor cells.** Primary NSCLC tumor cells were treated as previously described in **Fig. 4**. Immunofluorescence microscopy images taken at 10-fold magnification. Hoechst 33342 used to stain DNA (blue) and therefore identify the number of cells present in each well. Pan-cytokeratin antibody used to identify epithelial/tumor cells (parent population). From the parent population the cytochrome c positive cells % was determined using cytochrome c-647 antibody (red). DMSO treatment is a negative control for cytochrome c loss. Non-hit is a drug treatment that didn’t score a Z-score ≥ 3. Hit is the drug treatment that scored Z-score ≥ 3. The top hit is the highest Z-score for that primary NSCLC sample. Scale bar is 100 um.
